# Combined endoscopic lithotripsy and percutaneous transhepatic cholangioscopy therapy for refractory choledocholithiasis

**DOI:** 10.1055/a-2495-2744

**Published:** 2024-12-17

**Authors:** Kosuke Takahashi, Eisuke Ozawa, Hiroki Yamane, Masanori Fukushima, Hisamitsu Miyaaki, Kazuhiko Nakao

**Affiliations:** 1 34823 Third Department of Internal Medicine, University of Toyama, Nagasaki, Japan; 2 Department of Gastroenterology and Hepatology, Graduate School of Biomedical Sciences, Nagasaki University, Nagasaki, Japan; 3 Department of Gastroenterology and Hepatology, St Francis Hospital, Nagasaki, Japan; 4 Department of Gastroenterology and Hepatology, Sasebo City General Hospital, Nagasaki, Japan

A 39-year-old man who had undergone choledochojejunostomy with Roux-en-Y reconstruction for congenital biliary dilatation was referred to our hospital with choledocholithiasis. Computed tomography revealed large bile duct stones (BDS) in the hilum ( Fig. 1 **a** ). BDS removal was attempted using a double-balloon enteroscope (DBE); however, it failed to crush the BDS, especially those in the right hepatic duct branch ( Fig. 1 **b** ). Percutaneous transhepatic choledochoscopic electrohydraulic lithotripsy was performed; however, complete stone clearance was not achieved because of the limited range of motion of the cholangioscope caused by the BDS. Therefore, combined therapy comprising endoscopic lithotripsy and percutaneous transhepatic cholangioscopy was planned to maximize the effects of cholangioscopy with the support of the DBE ( Fig. 2 , Video 1 ).

**Fig. 1:**
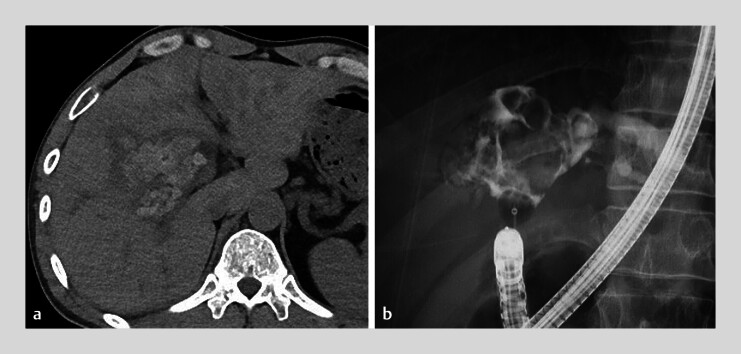
Images showing multiple large bile duct stones in the hilum. **a** Computed tomography. **b** Cholangiography.

**Fig. 2:**
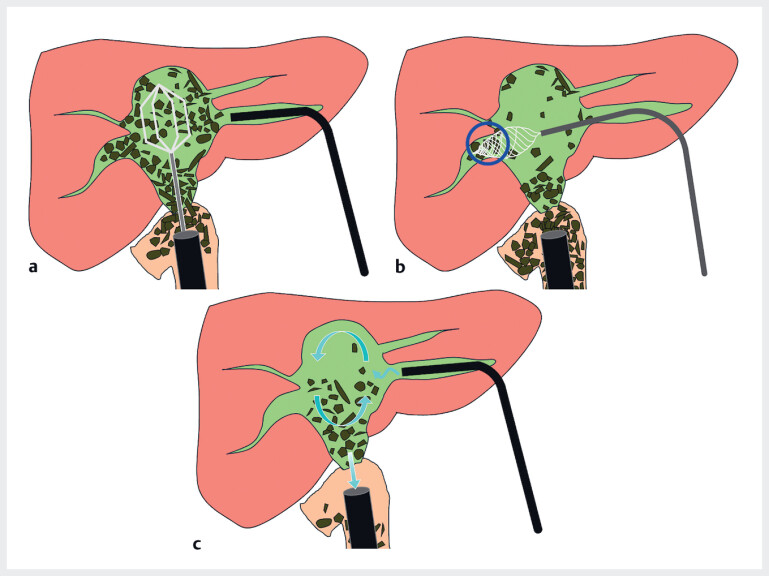
Schematic diagram of combined approach. **a** Endoscopic lithotripsy under cholangioscopic monitoring. **b** Percutaneous transhepatic cholangioscopy for bile duct stones for which endoscopic lithotripsy was difficult (circle). **c** Efficient stone removal through simultaneous water delivery from the cholangioscope and absorption via the endoscope.

Combined therapy comprising endoscopic lithotripsy and percutaneous transhepatic cholangioscopy for refractory choledocholithiasis.Video 1

Endoscopic stone removal was performed under cholangioscopic guidance. Subsequently, percutaneous transhepatic cholangioscopy was attempted for BDS that could not be removed by endoscopic lithotripsy; however, multiple small residual stones were identified during cholangiography. Therefore, stone removal from the anastomosis to the jejunum was performed using the force of water distributed from the biliary speculum and endoscopic aspiration. The BDS were efficiently removed from the anastomosis, and complete stone clearance was achieved ( Fig. 3 ).

**Fig. 3:**
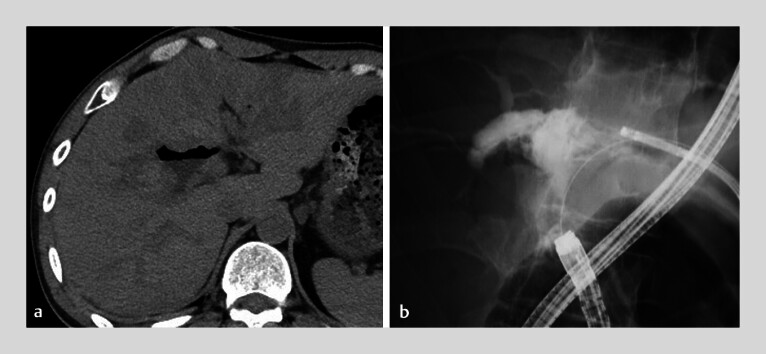
Images showing complete stone clearance. **a** Computed tomography. **b** Cholangiography.

Although percutaneous transhepatic choledochoscopic electrohydraulic lithotripsy is a useful treatment for refractory BDS 1 2 3 , stone clearance can be difficult when the working space for the cholangioscope is limited by large stones. An advantage of combined therapy comprising endoscopic lithotripsy and percutaneous transhepatic cholangioscopy is that different approaches using the endoscopic and percutaneous routes during the same session can performed in difficult areas, thereby reducing the procedure time. This method allows efficient stone removal using water delivery from the cholangioscope and water absorption via the DBE.

In conclusion, combined therapy comprising endoscopic lithotripsy and percutaneous transhepatic cholangioscopy is useful for refractory BDS.

Endoscopy_UCTN_Code_TTT_1AR_2AH

## Notes

Endoscopy E-Videos https://eref.thieme.de/e-videos
*E-Videos* is an open access online section of the journal *Endoscopy* , reporting on interesting cases and new techniques in gastroenterological endoscopy. All papers include a high-quality video and are published with a Creative Commons CC-BY license. Endoscopy E-Videos qualify for HINARI discounts and waivers and eligibility is automatically checked during the submission process. We grant 100% waivers to articles whose corresponding authors are based in Group A countries and 50% waivers to those who are based in Group B countries as classified by Research4Life (see: https://www.research4life.org/access/eligibility/ ). This section has its own submission website at https://mc.manuscriptcentral.com/e-videos .

## References

[1] Binmoeller KF Brückner M Thonke F Treatment of difficult bile duct stones using mechanical, electrohydraulic and extracorporeal shock wave lithotripsy Endoscopy 1993 25 201 206 10.1055/s-2007-1010293 8519238

[2] Mo LR Hwang MH Yueh SK Percutaneous transhepatic choledochoscopic electrohydraulic lithotripsy (PTCS-EHL) of common bile duct stones Gastrointest Endosc 1988 34 122 125 10.1016/s0016-5107(88)71276-6 3366328

[3] Kurihara T Yasuda I Isayama H Diagnostic and therapeutic single-operator cholangiopancreatoscopy in biliopancreatic diseases: prospective multicenter study in Japan World J Gastroenterol 2016 22 1891 1901 10.3748/wjg.v22.i5.1891 26855549 PMC4724621

